# International tuberculosis research collaborations within Asia

**DOI:** 10.1186/s13104-017-2769-4

**Published:** 2017-09-07

**Authors:** James S. Molton, Shweta Singh, Ling Jun Chen, Nicholas I. Paton

**Affiliations:** 1 Division of Infectious Diseases, University Medicine Cluster, National University Health System, 1E Kent Ridge Road, NUHS Tower Block, Level 10, 119228 Singapore, Singapore; 2 Department of Medicine, Yong Loo Lin School of Medicine, National University of Singapore, Singapore, Singapore

**Keywords:** Tuberculosis, Research, Asia

## Abstract

**Background:**

Asia bears more than half the global tuberculosis (TB) burden. Economic development in the region has increased available funding for biomedical research and opportunity for collaboration. We explored the extent of international tuberculosis research collaborations between institutions within Asia.

**Methods:**

We conducted a Pubmed search for all articles with tuberculosis in the title published during a 12 month period with at least one author affiliation listed in Asia, then identified international collaborations from institution websites and internet searches.

**Results:**

We identified 99 international collaborations involving an institution within Asia, of which only 8 (8.1%) were collaborations between Asian institutions. The remainder were with institutions outside of Asia.

**Conclusions:**

The paucity of intra-Asian international research collaboration represents a lost opportunity to optimise regional research funding, capacity building and the development of an Asia-relevant TB research agenda.

## Background

Asia has 58% of the global burden of TB and eight of the top ten high burden Multi-Drug Resistant TB countries [1]. Many countries in Asia have enjoyed periods of rapid economic growth in recent years and are now classified as middle/upper income countries [2]. Asian biomedical research investment tripled between 2004 and 2012 [3]. A number of respected Asian institutions have done significant TB research. Although there is enormous potential for international intra-Asian research collaboration, it was the perception of the authors that this was not occurring and we set out to examine this.

## Methods

We identified institutions in Asia with a TB research program by performing a Pubmed search (17th April 2013) for all articles that contained the word “tuberculosis” in the title, had at least one institutional affiliation in Asia and were published between 1st April 2012 to 1st April 2013. For the purposes of this report “Asia” includes all countries in the WHO South-East Asia Region plus those in the WHO Western Pacific Region that lie in continental Asia (i.e. excluding Australasia and the Pacific nations) [4]. A list of Asian institutions with at least one TB research article was compiled. We identified international collaborations by performing a search of the institution website for evidence of TB collaborations originating from that institution and by a Google search of the institution name and the word “tuberculosis”. Collaborations within the same country in Asia were not included, in order to ensure a balanced comparison with the collaborations outside Asia (which by definition are all international).

Collaborations were classified as “Clinical”, “Basic Science” or “Other”. Collaborations could be in more than one category. Within Clinical, collaborations were defined as: Clinical Trials; Clinical Observational Studies or Epidemiology. Within Basic Science, collaborations were defined as: Pathogenesis; Diagnostics; Vaccine Development or Drug Development, depending on the focus of the collaboration. Training and Logistical support collaborations were defined as any collaboration to support research but not directly involving research. Funding sources were not considered collaborating institutions unless there was evidence of direct academic involvement.

## Results

**Fig. 1:**
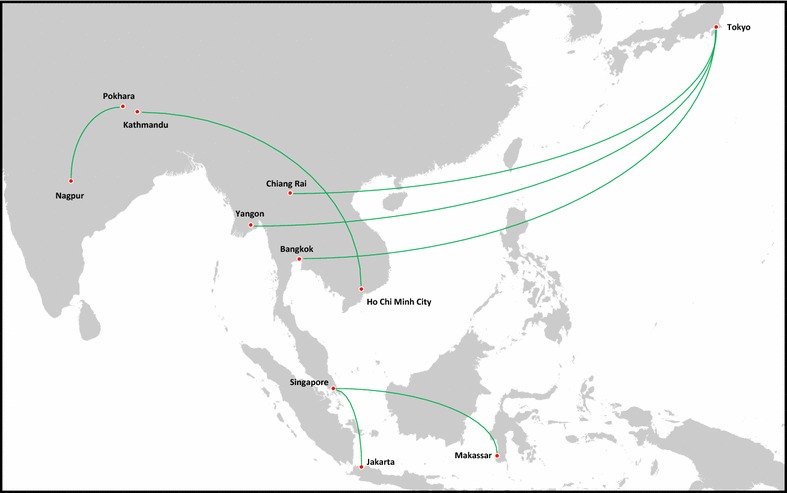
Tuberculosis related collaborations amongst institutions within Asia. It should be noted that there may be more than one collaborating institution in each city marked on the maps. List of countries included in “Asia” for the purposes of the Pubmed search: Bangladesh, Bhutan, Brunei Darussalam, Cambodia, China (inc. Hong Kong), India, Indonesia, Japan, Democratic People’s Republic of Korea, Republic of Korea, Lao People’s Democratic Republic, Malaysia, Maldives, Myanmar (Burma), Nepal, Papua New Guinea, Philippines, Singapore, Sri Lanka, Taiwan, Thailand, Timor-Leste, Vietnam. Maps produced using Flightmap software version 2.2.16 (liquidFOLDERS, Germany), reproduced with permission

**Fig. 2:**
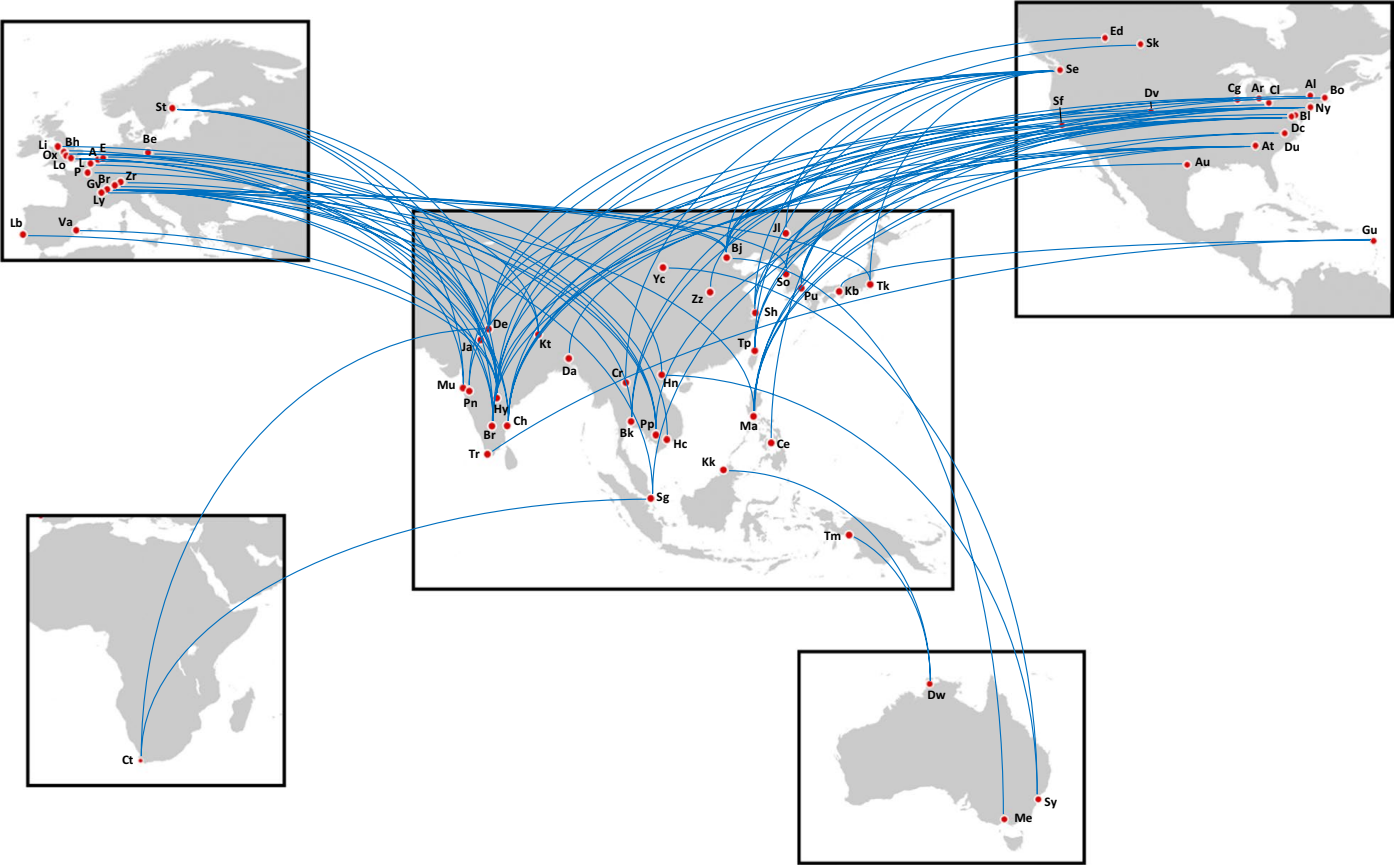
Tuberculosis related collaborations between institutions within Asia, and institutions outside Asia (United Kingdom: Li, Liverpool; Bh, Birmingham; Ox, Oxford; Lo, London; France: P, Paris; L, Lille; Ly, Lyons; Belgium: A, Antwerp; Holland: E, Eindhoven; Switzerland: Gv, Geneva; Br, Bern; Zr, Zurich; Spain: Va, Valencia; Portugal: Lb, Lisbon; Sweden: St, Stockholm; Canada: Ed, Edmonton; Sk, Saskatoon; USA: Sf, Stanford; Dv, Denver; Cg, Chicago; Ar, Ann Arbor; Cl, Cleveland; Al, Albany; Bo, Boston; Ny, New York; Bl, Baltimore; Dc, Washington D.C.; Du, Durham; At, Atlanta; Au, Austin; Guadeloupe: Gu, Guadeloupe; China: Jl, Jilin; Bj, Beijing; Yc, Yinchuan; Zz, Zhengzhou; Sh, Shanghai; Taiwan: Tp, Taipei; Republic of Korea: So, Seoul; Pu, Pusan; Japan: Kb, Kobe; Tk, Tokyo; Philippines: Ma, Manila; Ce, Cebu; Indonesia: Tm, Timika; Singapore: Sg, Singapore; Vietnam: Hc, Ho Chi Minh City; Hn, Hanoi; Cambodia: Pp, Phnom Penh; Thailand: Bk, Bangkok; Cr, Chiang Rai; Bangladesh: Da, Dhaka; Nepal: Kt, Kathmandu; India: De, Delhi; Ja, Jaipur; Mu, Mumbai; Pn, Pune; Hy, Hyderabad; Ch, Chennai; Br, Bangalore; Tr, Thiruvananthapuram; South Africa: Ct, Cape Town; Australia: Dw, Darwin; Me, Melbourne; Sy, Sydney). Maps produced using Flightmap software version 2.2.16 (liquidFOLDERS, Germany), reproduced with permission

Of 3439 articles published from 1st April 2012 to 1st April 2013 with the word tuberculosis in the title, 1082 listed at least one affiliated institution in Asia. The search of these affiliating institutions websites and further Google searches identified 66 institutions in Asia that had evidence of a TB research collaboration with one or more international institutions (within or outside Asia). These 66 organisations were involved in 99 cross-border collaborations, of which 8 were in Asia, 47 in North America, 36 in Europe, 6 in Australasia and 2 in Africa. The geographic distribution of Asian institutions involved in international TB collaboration was as follows: 23 institutions in India, 12 in China, 4 in Vietnam, 3 in Nepal, 3 in the Philippines, 3 in Thailand, 3 in Indonesia, 3 in Japan, 3 in Republic of Korea, 2 in Cambodia, 2 in Singapore, 2 in Taiwan, 1 in Bangladesh, 1 in Malaysia and 1 in Myanmar. These sites collaborated with institutions in the following countries outside Asia: 20 institutions in the USA, 5 in Switzerland, 5 in UK, 3 in France, 3 in Australia, 3 in Sweden, 2 in Canada, 2 in Germany, 2 in South Africa, 1 in Belgium, 1 in the Netherlands, 1 in Guadeloupe, 1 in Portugal and 1 in Spain. Of the total of 99 individual collaborations identified, 8 were collaborations between Asian institutions and 91 were with institutions outside Asia (Figs. 1, 2). Of the 8 collaborations within Asia, 2 involved clinical trials, 2 involved observational studies, 4 involved epidemiology, 2 involved pathogenesis studies, 2 involved diagnostics, 1 involved drug development, and 1 involved training and logistical support. Of the 91 collaborations between Asian and non-Asian institutions, 17 involved clinical trials, 26 involved observational studies, 10 involved epidemiology, 18 involved pathogenesis studies, 15 involved diagnostics, 5 involved vaccine development, 23 involved drug development, and 11 involved training and logistical support. Note each collaboration may span more than one category. A breakdown of research collaborations by category is shown in Table 1.

**Table 1:** Research collaborations by category

	Collaborations between Asian institutions	Collaborations between Asian and non-Asian institutions	Overall
Clinical			
Clinical Trials	2	17	19
Observational studies	2	26	28
Epidemiology	4	10	14
Basic science			
Pathogenesis	2	18	20
Diagnostics	2	15	17
Vaccine development	0	5	5
Drug development	1	23	24
Other			
Training and logistical support	1	11	12
Total number of collaborations	8	91	99

Note that each collaboration may consist of more than one category

## Discussion

We identified a large number of Asian institutions with investigators actively contributing to the TB research literature, as expected given the heavy burden of TB in this region. Most identified collaborations were with institutions outside Asia, predominantly in North America (47.5%) and Europe (36.4%), with a striking paucity of such collaborations within Asia (8.1%). Our search was limited to 1 year of published articles, which was able to provide a representative snapshot, but the list of collaborations identified is unlikely to be exhaustive. While it is unlikely that any major institutions engaged in TB research failed to publish a single paper during that period, collaborative research programs in their early stages may have yet to published any papers and might have been missed. Some research collaborations may not have been reflected on institutional websites, and it possible that there was a bias for Asian institutions to report international collaborations but omit regional collaborations if the former are perceived to be more prestigious, or are better funded. Some institution websites had not been updated for many years and it is possible that more recent collaborations were not detected. Training and logistical support collaborations were identified during the search process but the list is likely incomplete as these would often not result in publication.

In spite of these limitations, the overall finding is likely to be robust: that there is both a relative and absolute paucity of intra-Asian international research collaboration. The reasons for this are likely multiple, but the principal one is likely to be funding. Researchers in North America or Europe have access to funding from agencies such as the National Institute of Health, UK Medical Research Council and Wellcome Trust that encourage or sometimes require that funding is spent internationally in resource-limited settings, and allow this in part to support capacity building overseas [5–7]. In contrast, funding agencies in Asian countries, even those with a GDP higher than many Western countries, usually require that research funding is allocated exclusively to projects conducted within national boundaries, which reduces the incentive for regional networking and acts as a barrier to initiating international intra-Asian collaboration [8]. A second factor promoting collaborations outside Asia is the existence of historical ties (usually with a common language) that are often accompanied by educational opportunities such as PhD programmes that naturally perpetuate these links. Diversity of language may limit the opportunities for intra-Asian collaboration. We identified some collaborations between institutions within individual Asian countries, but did not count these in the current analysis because our focus was on international (cross-border) collaboration. Intra-country collaborations are likely to easier to establish and maintain, but nevertheless require a degree of coordination that in some cases may provide a basis for expanding to international collaboration.

This paucity of intra-Asian collaboration represents a lost opportunity in several ways. Firstly, according to the Treatment Action Group there is currently a major deficit in research funding identified across all aspects of TB research [9]. Many Asian countries regard TB as a national research priority and have the potential to allocate significant resources to this area. Greater collaboration across Asia may succeed both in unlocking additional national sources of funding but may also allow existing smaller quantities of funding to be combined more efficiently to conduct common research protocols that can address questions of high impact and generalisability across all participating countries. Secondly, there is a well-recognised deficit in global TB research capacity, especially to conduct the clinical trials needed to evaluate novel TB drugs and treatment regimens [10]. Given the existing high-level research skills that exist in some institutions within Asia, increasing intra-Asian research collaboration could represent an efficient means of research capacity building. Thirdly, the lack of strong intra-Asian collaboration means that an Asian perspective may be under-represented in the development and prioritisation of the TB research agenda.

A useful step to address the paucity of intra-Asian research collaboration would be to establish an Asian TB research network that can broker the use of regional funding, coordinate research capacity building and training (through observorships or formal PhD programmes) and bring together the expertise within the region to develop an Asia-focused research agenda.

## Conclusions

We identified a lack of intra-Asian TB research collaborations, which represents a lost opportunity to optimise regional research funding, capacity building and the development of an Asia-relevant TB research agenda.

## Abbreviations

GDP: gross domestic product
PhD: Doctor of Philosophy
TB: tuberculosis
WHO: World Health Organization
